# Exploration of pathomechanisms triggered by a single-nucleotide polymorphism in titin's I-band: the cardiomyopathy-linked mutation T2580I

**DOI:** 10.1098/rsob.160114

**Published:** 2016-09-28

**Authors:** Julius Bogomolovas, Jennifer R. Fleming, Brian R. Anderson, Rhys Williams, Stephan Lange, Bernd Simon, Muzamil M. Khan, Rüdiger Rudolf, Barbara Franke, Belinda Bullard, Daniel J. Rigden, Henk Granzier, Siegfried Labeit, Olga Mayans

**Affiliations:** 1 Department of Integrative Pathophysiology, Medical Faculty Mannheim, Theodor-Kutzer-Ufer 1-3, 68167 Mannheim, Germany; 2 Institute of Integrative Biology, University of Liverpool, Crown Street, Liverpool, L69 7ZB, UK; 3 Department of Cellular and Molecular Medicine and Sarver Molecular Cardiovascular Research Program, University of Arizona, Tucson, AZ 85724, USA; 4 School of Medicine, University of California San Diego, 9500 Gilman Drive, MC-0613C, La Jolla, CA 92093, USA; 5 European Molecular Biology Laboratory, Structural and Computational Biology Unit, Meyerhofstrasse 1, 69117 Heidelberg, Germany; 6 Institute of Molecular and Cell Biology, Mannheim University of Applied Sciences, Paul-Wittsackstraße 110, 68163 Mannheim, Germany; 7 Institute of Toxicology and Genetics, Karlsruhe Institute of Technology, Hermann-von-Helmholtz-Platz 1, 76344 Eggenstein-Leopoldshafen, Germany; 8 Department of Biology, University of York, York YO10 5DD, UK; 9 Department of Biology, University of Konstanz, 78457 Konstanz, Germany

**Keywords:** cardiomyopathy, missense single-nucleotide polymorphism, titin protein structure, transgenic muscle, transgenic mouse model

## Abstract

Missense single-nucleotide polymorphisms (mSNPs) in titin are emerging as a main causative factor of heart failure. However, distinguishing between benign and disease-causing mSNPs is a substantial challenge. Here, we research the question of whether a single mSNP in a generic domain of titin can affect heart function as a whole and, if so, how. For this, we studied the mSNP T2850I, seemingly linked to arrhythmogenic right ventricular cardiomyopathy (ARVC). We used structural biology, computational simulations and transgenic muscle *in vivo* methods to track the effect of the mutation from the molecular to the organismal level. The data show that the T2850I exchange is compatible with the domain three-dimensional fold, but that it strongly destabilizes it. Further, it induces a change in the conformational dynamics of the titin chain that alters its reactivity, causing the formation of aberrant interactions in the sarcomere. Echocardiography of knock-in mice indicated a mild diastolic dysfunction arising from increased myocardial stiffness. In conclusion, our data provide evidence that single mSNPs in titin's I-band can alter overall muscle behaviour. Our suggested mechanisms of disease are the development of non-native sarcomeric interactions and titin instability leading to a reduced I-band compliance. However, understanding the T2850I-induced ARVC pathology mechanistically remains a complex problem and will require a deeper understanding of the sarcomeric context of the titin region affected.

## 1. Introduction

Cardiovascular disease is the major cause of death worldwide. Evidence accumulated over the past two decades has identified titin as a main coordinator of cardiac muscle homeostasis, and its dysfunction through genetic mutation as an important factor in cardiomyopathy. Titin mutations have now been linked to familial dilated cardiomyopathy (DCM), hypertrophic cardiomyopathy (HCM) and arrhythmogenic right ventricular cardiomyopathy (ARVC) (recently reviewed in [1]). Pathogenicity often results from mutations that lead to the truncation of the titin chain, as identified in 25% of familial cases of idiopathic DCM and 18% of sporadic cases [2]. Several missense single-nucleotide polymorphisms (mSNPs) in titin have also been associated with heart disease. These comprise the ARVC-linked T2850I in the I-band [3]; the DCM-associated V54M, A743V in the Z-disc [4] and W930R in the Z-disc/I-band transition zone [5]; R740L [6] and S3799Y [7] that increase binding to α-actinin and FHL2, respectively, causing HCM; and Y7621C [8] located in the A/I-band junction linked to restrictive cardiomyopathies. Consequently, genetic screening of the titin gene (*TTN*) is clinically relevant in most cardiomyopathy cases [1,9]. However, while the robust discrimination between benign and disease-causing titin truncations can be achieved by coupling *TTN* exon inclusion data and the position of the mutation within the gene [10], predicting the pathogenicity of mSNPs in titin is considerably more complex. In contrast to truncations, mSNPs might result in individual molecular phenotypes and appear to trigger distinct pathomechanisms [11]. As a result, the differentiation between benign and pathogenic mSNP variants is currently very demanding, requiring the integration of functional assays, robust bioinformatics, large control cohorts and expert clinical evaluation [9].

In this work, we set to answer the question of whether single mSNPs in titin can generically and without the contribution of additional genetic factors lead to cardiac disease. For this, we examined the unique mSNP T2850I in the I-band domain of titin I10, as a paradigm of an alteration in a general component of the titin chain. Domain I10 does not support specific interactions or have any known specialized roles, so it does not constitute an *a priori* sensitive locus of the chain. The T2850I exchange has been associated with ARVC based on linkage studies, being completely segregated with the ARVC phenotype in nine patients from a large family, including two fifth-degree relatives, and was absent in 300 cardiomyopathy and 400 control chromosomes [3]. ARVC is characterized by life-threatening arrhythmias, being the main cause of sudden death in the population below 25 years of age. Mechanistically, ARVC is thought to result from a perturbed desmosomal force transmission [12]. Titin, however, is an intrasarcomeric protein not known to be related to cell adhesion. Thus, we asked whether mSNPs in titin can induce an ARVC phenotype. In previous studies, we have shown that the T2580I mSNP destabilizes the affected immunoglobulin (Ig) domain in titin, I10, and speculated that this instability increases the vulnerability of titin to proteolysis *in situ* potentially leading to myocardial damage [3,13]. Here, we study the dysfunction caused by this mutation by implementing an integrative approach from the protein domain to the whole organism. Results indicate that, in addition to fold instability, the mSNP alters the chemical reactivity of titin leading to the formation of non-native interactions in the sarcomere. Even though the molecular and cellular effects of the exchange are mild, they appear to lead to a detectable alteration of the diastolic behaviour of the heart in knock-in (KI) mice. Thereby, our results establish that a single SNP in a common Ig component of titin can disturb the overall performance of the heart in the absence of other genetic factors. The result points to a high potential of titin mSNPs for causing cardiac disease.

## 2. Material and methods

T2850 is encoded by the ACC triplet on chromosome 2 : 178769890–178769893 referenced to GRCh38/hg38 human genome assembly. This is mutation T2896I in Taylor *et al.* [3] and mutation T16I in Anderson *et al.* [13].

For *in vitro* studies, I9–I11 (residues 2749–3009) and I10 (2835–2895) were expressed recombinantly in *Escherichia coli* and purified to homogeneity by chromatography. For NMR studies, I10 was produced in M9 medium with ^15^NH_4_Cl as source of nitrogen and ^13^C-glucose as carbon source.

I10 crystals were grown in 0.2 M CaCl_2_, 0.1 M Tris pH 7.5 with either (a) 30% (w/v) PEG 3350, 3% (v/v) isopropanol or (b) 25% (w/v) PEG 8000 as precipitants. Crystal (a) yielded a crystallographic model to 2.00 Å resolution with an *R*_factor_/*R*_free_ = 17.53/22.99%. Crystal (b) produced a model to 1.8 Å and *R*_factor_/*R*_free_ = 17.45/20.49%. I19–I11 crystals were produced in (c) 0.1 M Tris HCl pH 8.5, 30% (w/v) PEG 4000, 0.2 M MgCl_2_; (d) 0.1 M Bis-Tris propane pH 8.5, 20% (w/v) PEG 3350, 0.2 M sodium acetate. A three-dimensional model from (c) was to 1.9 Å with *R*_factor_/*R*_free_ = 19.28/22.83%; and from (d) to 1.53 Å with an *R*_factor_/*R*_free_ = 16.15/19.65%. Structures have been deposited with the Protein Data Bank with access codes: 4QEG, 5JDJ, 5JDE and 5JDD.

For molecular dynamic simulations, the crystal structure of I10–I11 was used as wild-type and the T2850I modelled *in silico*. A ‘protein in a box of water’ simulation of 50 ns followed standard protocols in GROMACS 5.0. Principal component analysis was used to analyse the trajectories for differences.

All animal experiments were approved by local ethics committees. T2850I mutation carrying mice were generated using gene-targeting. Genotype was confirmed by direct sequencing. The mice were on a mixed background, 50% 129S6 and 50% C57BL/6. Echocardiography was performed under isoflurane anaesthesia using a Vevo 2100 High-Resolution Imaging System and accompanying software.

GFP-tagged titin fragments were introduced into *tibialis anterior* muscle using electroporation under general anaesthesia. Mice were sacrificed 10 days later and transfected muscle sections visualized by laser scanning confocal microscopy for localization of titin fragments or differential centrifugation of muscle extracts was also performed. Tissue was fractionated into cytosolic, microsomal and particulate fractions and titin fragments were detected by western blotting using anti-GFP antibody.

Neonatal mouse cardiomyocytes were isolated from 1- to 3-day-old pups. Cells were transfected with GFP-tagged titin fragments and fixed 48 h later. Cells were counterstained with antibodies against α-actinin, filamentous actin and DNA and imaged by confocal microscopy.

A comprehensive description of material and methods is provided in the electronic supplementary material.

## 3. Results

### 3.1. The T2850I mutation strongly destabilizes domain I10

To estimate the effect of the T2850I exchange on the fold stability of I10, the thermal denaturation of wild-type and mutated samples (I10^WT^ and I10^T2850I^, respectively) was monitored using differential scanning fluorimetry (DSF) (electronic supplementary material, section S1). The melting temperature (*T*_m_) of I10^WT^ was derived from a single transition melting profile as 62.3 ± 1.3°C, in good agreement with its *T*_m_ value previously calculated from circular dichroism data (60.1 ± 0.2°C) [14]. This value indicates that I10 is an intrinsically stable domain. The *T*_m_ value of I10^T2850I^ was 51.4 ± 1.8°C, approx. 11°C below that of I10^WT^. Comparably acute Δ*T*_m_ decreases in titin Ig domains have only been observed when drastic truncations or modifications of the fold occurred. Accordingly, our measurement of a truncated version of I10 missing the N-terminal β-strand A showed a Δ*T*_m_ ≈ −14°C (electronic supplementary material, figure S1) and the deletion of just four residues in other Igs of titin decreased their *T*_m_ values by up to 15°C [15,16]. A similar ΔT_m_ decrease was observed in domain Z1 when the CD loop was replaced in its totality for an exogenous sequence that eliminated β-strand C’ and, thereby, removed the native capping of the Ig β-sandwich fold [17]. These data indicate that the impact of the single-point mutation T2850I on I10 is equivalent to that of a large insult on the fold, such as removal of β-strand A or the CD loop. This significant destabilizing effect suggests that T2850 plays an important structural role in I10.

### 3.2. The crystal structure of I10 reveals a high-energy conformation for residue T2850

**Figure 1.**
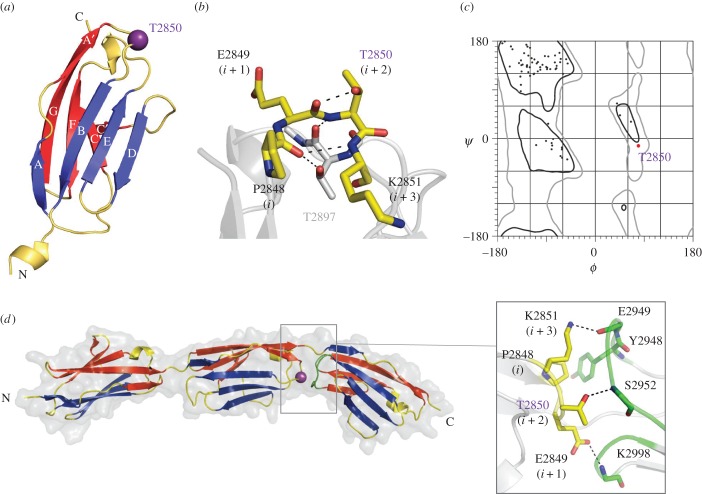
Crystal structure of I10 in isolation and in the context of the I9–I11 tandem. (*a*) Representation of I10^WT^. The component β-sheets (A'FCC'G and ABED) are coloured in red and blue, respectively. Residue T2850 in the A'B β-turn is indicated with a purple sphere; (*b*) structural features of the A'B β-turn of I10^WT^. Hydrogen bonds are indicated by dashed lines; (*c*) Ramachandran plot showing that the main chain conformation of T2850 is located in the generously allowed left-handed α-helical region (αL) (the electron density map of T2850 in electronic supplementary material, figure S2 shows that the conformation of this residue is well-defined experimentally); and (*d*) crystal structure of I9–I11 and detail of the I10 (yellow)–I11 (green) domain interface (inset).

To gain a molecular insight into the structural role of residue T2850, we elucidated the atomic structure of domain I10 at 1.74 Å resolution using X-ray crystallography (diffraction data statistics and model refinement parameters are given in the electronic supplementary material, table S2). We calculated a total of 17 molecular copies of I10 originating from two different space groups and diverse non-crystallographic symmetries. The structural features of all 17 molecular copies of I10 were in strict agreement (global RMSD = 0.62 Å for 82 matched Cα atoms across all models, calculated using MUSTANG [18]). The structures showed that I10 displays a classical Ig I-type fold (figure 1*a*) and that residue T2850 is located at the C-terminal pole of the fold, in position *i* *+* 2 of the β-turn connecting β-strands A’ and B (experimental electron density shown in the electronic supplementary material, figure S2). This is a β-turn type II, where residue *i* *+* 2 adopts a left-handed helix conformation (αL Ramachandran region) that is sterically restricted [19]. T2850 is stabilized in this conformation by two hydrogen bonds (figure 1*b*): (i) a short, strong bond (2.4 Å) between its side chain hydroxyl and the carbonyl of the previous residue, E2849, and (ii) a second contact between its main chain amide group and the main chain carbonyl of T2897 in the neighbouring EF loop. The side chain hydrogen bond turns the otherwise atomic clash into a productive interaction and permits T2850 to adopt a high-energy conformation within the generously allowed αL Ramachandran region (figure 1*c*).

A side chain with a branched or cyclic Cβ atom (such as that of residues Ile, Val and Pro) in position *i* *+* 2 could result in clashes with the carbonyl group of the preceding *i* *+* 1 residue and, thereby, be sterically unsatisfactory. To investigate this deduction, we carried out a study of 62 641 unique β-turns type II naturally occurring in proteins using PDBeMotif [20] (electronic supplementary material, table S3). We observed that position *i* *+* 1 is permissive to all residue types, but that position *i* *+* 2 strongly favours glycine (approx. 78% occurrence) with other amino acid types present at lower, but significant frequencies. In agreement with our expectations, amino acids with a branched Cβ-atom are very rare in position *i* *+* 2: isoleucine is present only in 2 out of 62 641 cases (3 × 10^−5^ occurrence), and valine in 22/62 641 (3.5 × 10^−4^). Proline, with a cyclic Cβ-atom, is the most uncommon residue at 1/62 641 cases (1.5 × 10^−5^). The few cases where proline or isoleucine residues were found in position *i* *+* 2 were examined manually and found to correspond to exceptionally rare turns in internal core positions, stabilized by rich hydrophobic contacts. Deductions from natural residue occurrence were in full agreement with calculations of the differential free energy (ΔΔG) of residue tolerance in I10 using FoldX [21], which also proposed Ile, Pro and Val as poorly tolerated residues at this locus of the I10-fold (electronic supplementary material, figure S3). Our conclusion is further supported by previous studies that evaluated amino acid energetics and compositional potential in type-II β-turns using small sample populations [19,22]. We further observed that threonine residues at this position invariably establish a tight hydrogen bond with the preceding main chain carbonyl group. This hydrogen bond is not present in β-turns II containing the Thr-resembling residues Ser and Cys, indicating that this is a mechanism to specifically stabilize the branched Cβ-atom of Thr in that position.

### 3.3. T2850I does not induce structural changes in I10 but increases its internal flexibility

**Figure 2.**
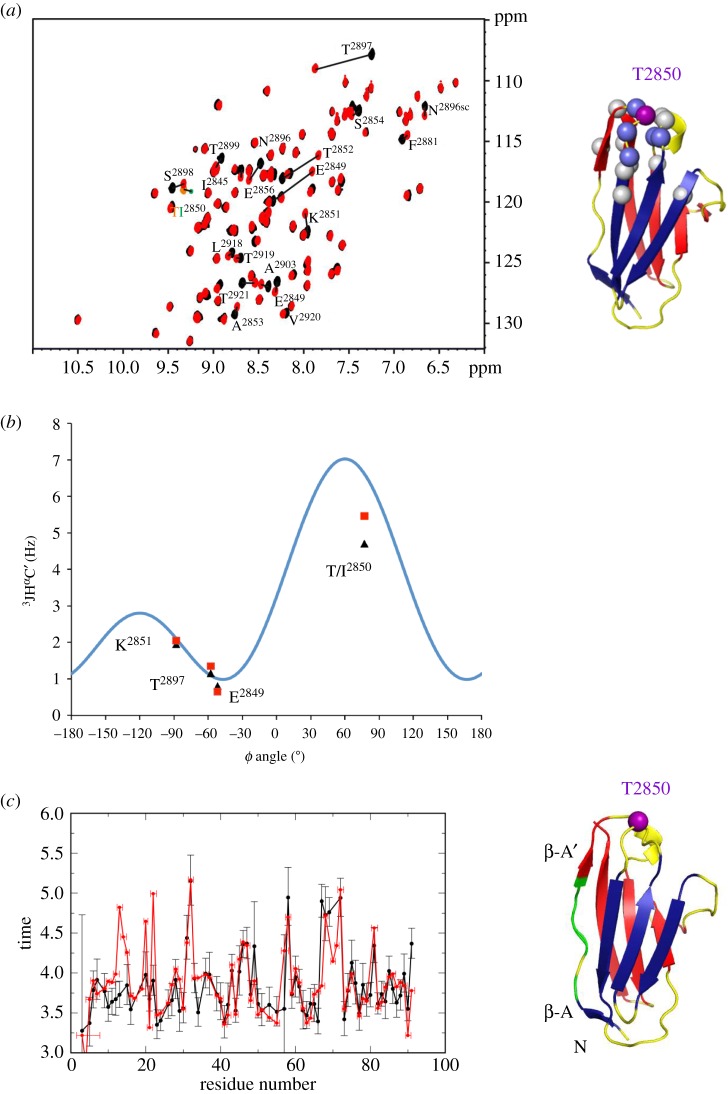
NMR analysis of changes in I10 induced by the T2850I exchange. (*a*) H^1^–N^15^ HSQC spectra (left). Residues affected by notable differences are indicated and mapped on the crystal structure of I10 (right). It can be seen that significant changes only occur in the immediate vicinity of the T2850I exchange. (*b*) Measured ^3^J(C’_k−1_Hα_k_) values plotted against the *ϕ* dihedral angle observed in the crystal structure of I10^WT^. The blue curve shows the dependency of the coupling constant upon the torsion angle *ϕ*. Both wild-type and mutant show large J-couplings for residue 2850 characteristic for positive *ϕ* angles. (*c*) Ratio of transverse (R2) and longitudinal (R1) ^15^N relaxation rates (left). Values above the average can be mapped to the A'B β-turn and β-strand A (right; green). Here and throughout this figure, values corresponding to I10^WT^ samples are in black and I10^T2850I^ in red.

The effect of the T2850I exchange on I10 was studied using NMR. H^1^-N^15^ HSQC spectra of both wild-type and mutated samples showed sharp, well-dispersed peaks characteristic of folded proteins, revealing that the overall fold is preserved in I10^T2850I^ (figure 2*a*). However, a fraction of amide resonances was perturbed by the mutation. Chemical shift perturbations were quantified using a weighted average difference [23] and regarded as ‘moderate’ (0.03 < Δδ_AV_ < 0.15 ppm) or ‘large’ (Δδ_AV_ > 0.15 ppm). They showed that in the 91 residue-long I10^T2850I^, 6 residues underwent large and 10 residues moderate chemical shift perturbations. When mapped onto the crystal structure of I10, the perturbations largely clustered around the mutation site, in the A'B β-turn and the neighbouring EF loop that are interconnected through hydrogen bonds mediated by T2850 (figure 2*a*). This shows that the mutation only introduces modest local distortions in the I10-fold. The characteristic backbone conformation of the A'B turn with a positive main chain dihedral *ϕ* angle in position *i* + 2 is best quantified by the measurement of the indirect coupling constant 3 J(C’_k−1_H_k_α). In this experiment, we observed similar couplings throughout the backbone of wild-type and mutant samples and especially the large J-values characteristic for the positive *ϕ* angles of both Thr and Ile residues (figure 2*b*). This indicated that the protein backbone conformations of both wild-type and mutant are highly similar and that the exchange does not notably affect the protein structure.

Next, we examined whether the T2850I mutation alters the internal dynamics of the protein by measurement of ^15^N relaxation data. The ratio of transverse (R2) and longitudinal (R1) relaxation rates gives a direct indication of the internal backbone dynamics of an amino acid. The average value of R2/R1 is determined by the overall rotational tumbling of the protein in solution and unaltered for most regions in the wild-type and mutant proteins (figure 2*c*). The outstanding exception are residues in the A'B β-turn and the preceding β-strand A: here we observed a significant increase in the R2/R1 ratios, which indicates that in the mutated protein these residues are involved in a slow (microsecond to millisecond timescale) structural exchange process between a wild-type like ground state and one (or several) altered conformations. Derivation of quantitative dynamical parameters using the S2-order parameters, which is an indicator of the fast timescale (picosecond to nanosecond), reveals that backbone dynamics are very similar for wild-type and mutant Ig10. Thus, it can be concluded that the T2850I exchange increases internal domain flexibility not only locally but that it has a knock-on effect on the preceding secondary structure, loosening the N-terminal fraction of the I10-fold, shown above to be important for the stability of I10.

### 3.4. The crystal structure of I9–I11 shows that T2850 mediates interdomain contacts

Residue T2850 is located at the C-terminal loop region of the Ig fold. To study whether this region is involved in interactions with the next Ig domain packed serially along the chain and, thus, whether the T2850I exchange influences the Ig-tandem architecture of titin, we determined the crystal structure of I9–I11 (comprising I10 in its poly-domain context) to 1.53 Å resolution. Three molecular copies were obtained in two space groups that agreed in showing I9–I11 in an extended conformation (figure 1*d*). Such extended arrangements are common in titin Ig-tandems [24,25]. In I9–I11, the component domains follow a regular arrangement, where domains display relative torsion angles of 45°–68° and are connected by short, two-residue sequences (TL and PI, respectively). In each Ig-pair, the domain interface is consistently formed by the A'B β-turn of the N-terminal domain slotting between the BC and FG loops of the C-terminal domain. Here, domains interact directly through a limited number of contacts (listed in the electronic supplementary material, section S2). Both I9–I10 and I10–I11 interfaces consist of a central hydrophobic residue contributed by the linker sequence (TL; PI), flanked by two polar interactions between loop residues from the neighbouring domains (figure 1*d*, inset). In addition, electrostatic potential maps of single domains in the I9–I11 tandem revealed that the N- and C-terminal poles of all domains are, respectively, positively and negatively charged. This suggests that an electrostatic component further assists the organization of these Ig domains within the titin chain.

Residue T2850 is buried within the I10–I11 interface (burial fraction of side chain is 0.79 as calculated with FoldX [21]). Here, T2850 preserves its intradomain hydrogen bonds (to T2897 and E2849) observed in the isolated I10 structure and, in addition, hydrogen bonds with S2952 from the BC loop of I11. This suggests that the exchange of the T2850 residue will cause a local alteration of the conformational dynamics of the titin chain.

### 3.5. The T2850I exchange alters the relative orientation of domains in the chain

As residue T2850 is involved in both the intra- and interdomain organization of titin, we studied next the effect of the T2850I exchange on the conformational dynamics of the I10–I11 tandem using molecular dynamics simulations (MDS) (electronic supplementary material, section S4). In total, 50 ns simulations were performed on the wild-type and on two T2850I models that corresponded to two different rotamers of the isoleucine residue. In addition, one wild-type and one T2850I variant were repeated with a different water model. The simulations were continued until no new conformational space was sampled as reflected by principal component analysis (electronic supplementary material, figure S4*b*–*d*). As a control that the time of the simulation was not limiting possible interdomain motions of larger amplitude, we performed a simulation on the Ig pair I65–I66 (extracted from PDB entry 3B4B [26]). In I65–I66, Ig domains are linked by a hydrophilic linker, three residues long, that allows unrestricted motions. This simulation confirmed that 50 ns simulation allows the sampling of multiple extreme conformations (electronic supplementary material, figure S4*a*).

**Figure 3.**
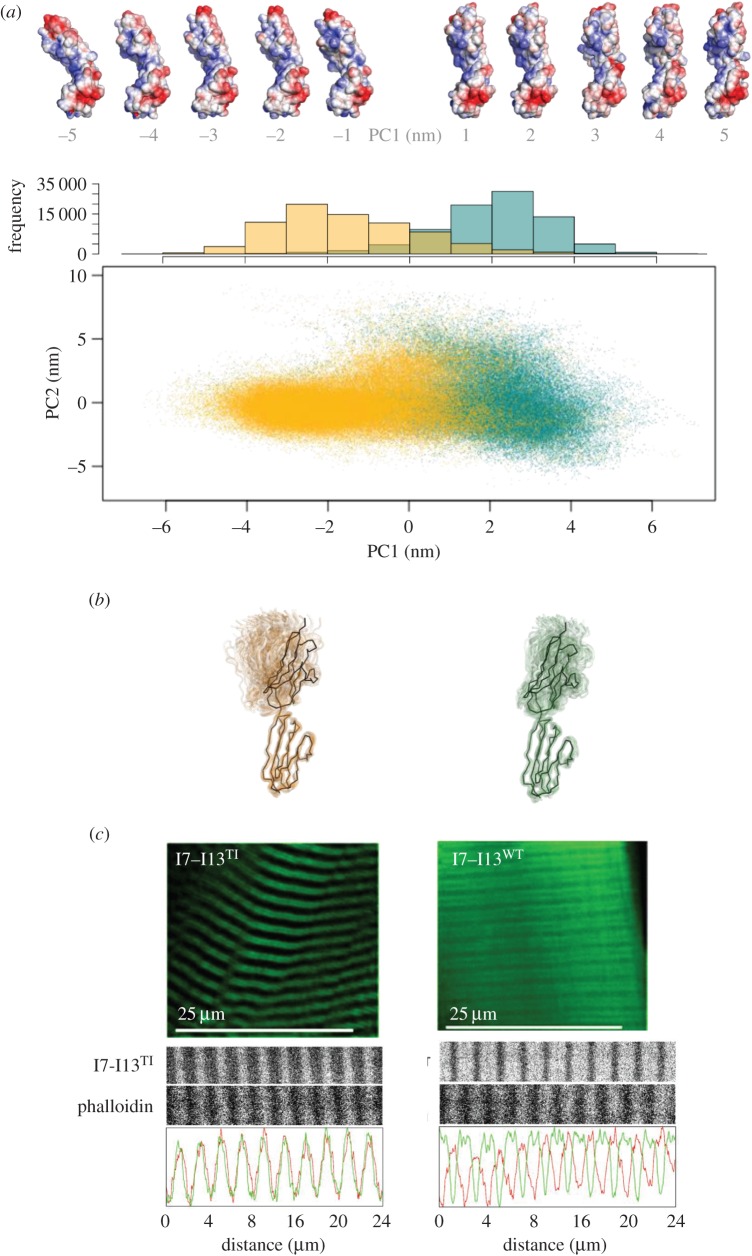
Different dynamic properties of wild-type and T2850I-mutated structures. (*a*) Molecular dynamics simulations of I10–I11. Top: electrostatic surface representation of conformational classes (using APBS [27]). Bottom: PC1 values plotted against PC2 values, showing static shift of relative conformations between trajectories with a histogram of PC1 eigenvalue frequency; (*b*) cloud cartoon representation of trajectory snapshots at 0.5 ps intervals. The initial crystal structure is shown as black ribbon. Throughout (*a,b*), T2850I is in gold and WT in green; (*c*) Differential localization *in vivo* of wild-type and mutant GFP-I7–I13 samples. Wild-type samples remained mostly diffused in the cytoplasm, with a weak association with the sarcomeric A-band as indicated by double-labelling with phalloidin. T2850I samples strongly interacted with the sarcomeric I-band co-localizing with the phalloidin stain.

Calculated trajectories for all I10–I11 models were converted to backbone models and concatenated, then subjected to covariance analysis. This showed that interdomain motions could be largely described by the first three principal component eigenvectors (PC1, PC2 and PC3), with PC1 and PC2 describing the largest movements. PC1 describes the rotational movement of I10 with respect to I11 (which totals a range of 68^o^), while PC2 describes the bending opening between I10 and I11 (spanning a range of 65^o^). When PC1 and PC2 values are plotted for all the trajectories, a difference in the conformational space sampled by wild-type and T2850I mutant is revealed—in particular, for the dominant component PC1 (figure 3*a,b*). Wild-type trajectories have more positive eigenvalues; in other words, inter-domain positions are aligned straight along an imaginary axis connecting the two domains' centres of mass, with both domains facing a similar starting direction. Conversely, the trajectories for T2850I mutants show negative eigenvalues that describe a greater degree of twisting, where the I10 and I11 modules face different directions. In combination with other components of movement, such as PC2's bending, this results in the T2850I mutants favouring a more bent and twisted conformation, that contrasts with the more extended arrangement preferred by the wild-type (figure 3*b*). This result suggests that wild-type and mutant favour somewhat different areas of conformational space, which might lead to titin acquiring different chemical surface properties at the I10–I11 locus upon mutation.

### 3.6. The T2850I exchange affects the myocellular reactivity of the titin Ig-tandem *in vivo*

To test whether the altered conformation of the T2850I-containing titin chain leads to an altered function *in vivo,* we assayed a GFP-tagged I7–I13 titin fragment in its wild-type and mutated versions in skeletal muscle of living mice. The fragment was introduced into the *tibialis anterior* muscle by electroporation and its expression monitored by western blot of muscle extracts using an anti-GFP antibody (electronic supplementary material, section S5). Single sharp protein bands corresponding to the expected molecular weight of I7–I13 were revealed this way. Signs of differential degradation of wild-type and mutated forms were not evident. This did not agree with a previous *in vitro* study, where recombinant I7–I13 and I7–I13^T2850I^ samples incubated with heart extracts indicated a notably reduced half-life of the mutated variant, probably as a result of proteolysis [13]. The findings in the current study suggest that the proteases that acted on the recombinant samples when extracts were used might not natively access cytoplasmic samples in the myofibril (e.g. due to compartmentalization) or, alternatively, that the proteolytic components of skeletal muscle are not representative of those from cardiac tissue. Either way, the results did not bring further support to *in situ* titin proteolysis as a mechanism of disease.

**Figure 4.**
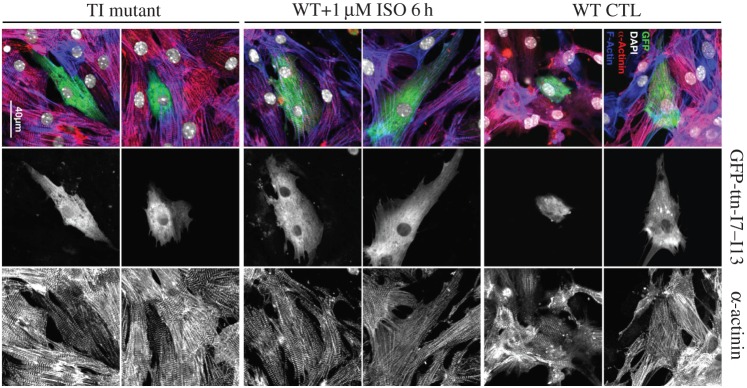
Expression of I7–I13 samples in neonatal cardiomyocytes. Z-discs were stained with α-actinin (right panel, red in the overlay), filamentous actin with fluorescent phalloidin (blue in the overlay), transfected titin fragments with EGFP (middle panel, green in the overlay) and nuclei with DAPI (white in the overlay). Both, wild-type (WT CTL) and mutant fragments of titin (TI) remain mainly diffuse in the cytoplasm of neonatal cardiomyocytes. No striated pattern compatible with sarcomeric targeting was observed, even in cells transfected with the wild-type construct that were challenged with 1 µM isoproterenol (ISO) for 6 h. Shown are two representative cells for each condition. Scale bar, 40 µm.

Imaging of the transfected muscles using *in vivo* confocal light microscopy revealed a different myocellular localization of wild-type and mutant I7–I13 (figure 3*c*). Wild-type fragments remained mostly soluble in the cytoplasm, associating only weakly with the sarcomere as suggested by the weak striated pattern. Fixed sections of the GFP-I7–I13 transgenic muscle that had been further stained with a fluorescent phalloidin conjugate (which binds F-actin forming the thin filaments), revealed that wild-type I7–I13 associated with the sarcomere in areas lacking F-actin, i.e. the H-zone of the A-band. As the native location of I7–I13 within the titin chain is the I-band, we concluded that the weak A-band patterning resulted from unspecific interactions due to the elevated expression levels. This result is not unexpected as previous studies with recombinant titin fragments did not detect binding of titin Ig-tandems to actin or other sarcomeric components [28,29]. By contrast, I7–I13^T2850I^ formed a well-defined striated pattern. Phalloidin staining showed that I7–I13^T2850I^ bands colocalized with F-actin, at the I-band. However, subcellular fractionation and actin co-sedimentation experiments showed that the binding was not to actin itself or other primary sarcomeric components (electronic supplementary material, section S5). Unfortunately, efforts to identify the interactor using pull-downs in muscle extracts and yeast two-hybrid screens were not successful. Contrary to the transgenic mouse muscle experiments, the transfection of mouse neonatal cardiomyocytes with I7–I13 and I7–I13^T2850I^ did not reveal localization differences (figure 4). Both fragments remained diffused in the cytoplasm under basal conditions and even after isoproterenol stimulation (electronic supplementary material, section S6). Neonatal cardiomyocytes have fully developed sarcomeres but lack T-tubules, which make us speculate that a T-tubule component might be the putative target of the pathological interaction. Nonetheless, as the I7–I13^T2850I^ sarcomeric pattern agrees with the expected native position of I7–I13, this pathologically increased affinity for an I-band component is likely to be physiologically relevant for this mSNP in its natural context within full-length titin.

### 3.7. Transgenic mice carrying the T2850I exchange present enhanced diastolic stiffness

To determine whether the mutation has functional effects at the organ level, we generated T2850I KI mice and performed echocardiography. No differences were found in left ventricular chamber dimensions during diastole and systole, ejection fraction or stroke volume (table 1). Pulse-wave Doppler echocardiography was used to measure the velocity of diastolic filling at the level of the mitral valve. Filling is known to occur in two waves: early diastolic filling (E-wave) and late diastolic filling due to atrial contraction (A-wave). The E/A ratio is the ratio of the early (E) to late (A) ventricular filling velocities and reflects diastolic function. The E/A ratio is used as an index for diastolic heart failure. Depending on the genetic background of the mouse strain, the E/A ratio in mice is 1.25–1.6. For example, in FVB mice after a two-week long transverse aortic constriction, which is a severe insult on the heart, the E/A ratio increases from 1.26 to 2.5 [30]. In T2850I KI mice, the E/A ratio was increased (from 1.67 in wild-type to 2.19 in the mutant) suggesting diastolic dysfunction. Furthermore, the E-wave deceleration time (DT) varies inversely with left ventricular diastolic stiffness and is another measure for the myocardial stiffening [31]. A significant E-wave DT reduction was found in T2850I KI mice, further supporting an increase in diastolic chamber stiffness. In conclusion, T2850I KI mice have a diastolic dysfunction.

**Table 1.** Cardiac parameters in T2850I transgenic mice. LVIDd: left ventricular internal diastolic diameter; WTd: diastolic wall thickness (average of posterior and anterior walls); LVIDs; left ventricular internal systolic diameter; WTs: systolic wall thickness (average of posterior and anterior walls); LV Vol; d: left ventricular diastolic volume; LV Vol; s: left ventricular systolic volume; LVW: left ventricular weight (mg); EF: ejection fraction; SV: stroke volume; MV E, mitral valve early diastolic peak filling velocity; MV A, mitral valve late diastolic peak filling velocity; MV Decel: deceleration time of E-wave (ms); MV E/A: ratio of MV E : MV A; LA: left atrium. *p*-value: significance value calculated with *t*-test; *p < 0.05 (indicated in bold).

	WT (*n* = 7)	T2850I (*n* = 7)	*p*-value
LVIDd (mm)	4.72 ± 0.14	4.78 ± 0.14	0.79
WTd (mm)	0.82 ± 0.01	0.84 ± 0.02	0.42
LVIDs (mm)	3.41 ± 0.14	3.54 ± 0.16	0.55
WTs (mm)	1.20 ± 0.04	1.21 ± 0.02	0.84
LV Vol;d (μl)	103.1 ± 6.6	106.8 ± 7.2	0.72
LV Vol;s (μl)	48.4 ± 4.5	54.1 ± 5.7	0.44
LVW	158 ± 6	172 ± 9	0.21
EF (%)	53.3 ± 2.9	49.9 ± 2.0	0.35
SV (μl)	54.8 ± 4.2	52.6 ± 2.6	0.67
MV E (mm s^−1^)	581 ± 32	647 ± 53	0.31
MV A (mm s^−1^)	351 ± 22	300 ± 26	0.15
MV Decel	30.3 ± 0.8	**26.5** **±** 1.1*	**0.017**
MV E/A	1.67 ± 0.08	**2.19** **±** 0.15*	**0.011**
E/E′	35.7 ± 2.9	35.6 ± 3.1	0.99
LA (mm)	3.07 ± 0.18	3.26 ± 0.24	0.54

## 4. Discussion

The genetic screening of the *TTN* gene, now pursued in large patient populations, has the potential to assist the diagnosis of cardiomyopathies, assess prognosis and guide therapy. However, identifying disease-causing missense alleles in titin is challenging, with the unexpectedly large number of rare genetic variants making their scoring a staggering task [32,33]. Rare sarcomeric gene variations associated with cardiomyopathy in small patient families were found in 17% of the NHLBI GO Exome Sequencing Project population [34], indicating that association studies alone are insufficient to evaluate the pathological potential of rare variants. In addition, rare genetic variants of sarcomeric genes often have complicated penetrance patterns. It was shown that the risk of adverse cardiovascular events grows with increasing numbers of rare sarcomeric variants [35]. Thus, methods are now urgently needed that can improve mSNP classification and unleash the wealth of information in *TTN* databases.

To gain an insight into the damage potential of mSNPs in titin—in particular, in the heart that appears to be more susceptible to mutations than skeletal muscle—we have studied the exchange T2850I in domain I10 of titin's I-band, which has been proposed to be linked to ARVC [3]. In this work, we went beyond our initial clinical and biophysical characterization of the mutation [3,13] and explored manifestations of this mSNP at different levels of biological complexity, ranging from molecular to organismal levels. In this work, atomic resolution three-dimensional structures of the affected titin region were generated as well as, to our knowledge, the first mouse model of a cardiomyopathy-associated titin mSNP. Our results show that the T2850I exchange only has a subtle impact on the titin molecule as well as at the tissue and animal levels. This was not anticipated, as it is intuitively expected that the severity of the disease promoted by an mSNP will correlate with the extent of the molecular damage that it causes. Structural data (HSQC and ^3^J(C’_k−1_H_k_α)) demonstrate that the T2850I exchange is tolerated by the I10-fold, not causing any detectable structural aberration. However, DSF data show that the mutated domain is notably less stable than the wild-type. The large difference in measured thermal stability was difficult to understand, as the exchange involves the removal of a single hydrogen bond (established by the Thr side chain with the main chain of the preceding residue; T2850-OH:CO-E2849) in the otherwise-intact fold. However, NMR relaxation data showed that the exchange not only increased the flexibility of the affected loop but also of the preceding β-strand A. This N-terminal segment of the fold is known to be of key importance for the chemical [26], thermal [15,16] and mechanical stability of the fold [36]. Consequently, our own measurement of an I10 variant lacking the N-terminal β-strand A showed a thermal destabilization comparable to that of the T2850I point mutant (electronic supplementary material, section S1). That the mutated Ig is also mechanically weaker has been established previously by atomic force microscopy [13], so that its unfolding is likely to occur at physiological muscle tension. The impact of an unfolded Ig on the overall elasticity of the titin chain seems negligible. However, it was originally proposed [13] that Ig unfolding (resulting from either stretch or spontaneous unfolding due to the intrinsic weakness of the mutated domain) might lead to *in situ* proteolysis of the titin chain, even though depletion of mutated titin from sarcomeres was not tested. In the current study, degradation of I7–I13 tandems expressed in muscle was not apparent, suggesting that I10 unfolding might have other functional consequences. A recent study has shown that induced unfolding of titin Ig segments in cardiomyocytes results in elevated stiffness caused by the aggregation of titin chains [37]. Yet, in diseased human muscle and heart, HSP27 or αβ-crystallin associates with the unfolded modules, preventing aggregation and suppressing the stiffening [37]. Hence, the extent to which I10 unfolding, specifically, contributes to the T2850I pathology is unclear.

The second consequence of the mutation is at the chain level, effected by its location in a domain junction. Our previous characterization of titin segments showed that the properties of domain interfaces are finely tuned along the chain, exhibiting conserved features in different parts of the sarcomere [24,25]. Not surprisingly, MDS suggests that the T2850I exchange influences the conformational dynamics of the titin chain at that locus, possibly altering its surface chemistry. An important deduction from this work is that mutations located at domain interfaces in titin can have an unexpected pathological potential, even when not causing major structural damage to the component domains. This finding is in agreement with our transgenic muscle data that point to the development of pathological binding capacity in the mutated titin's I-band *in situ*. Interestingly, the differential sarcomeric localization of wild-type and mutated samples could not be reproduced in neonatal cardiomyocytes and further efforts to identify the possible interactor of mutated titin failed. Upon differential centrifugation of transgenic muscles extracts, titin fragments were solely found in cytoplasmic and not in particulate myofibrillar or microsomal fractions, implying a fragile nature of the pathological interaction. Speculatively, we consider the possibility that the interaction might involve components of T-tubules as these are absent in neonatal cardiomyocytes.

Interspecies conservation of the affected residue allowed us to generate a knock-in mouse model carrying the titin T2850I mSNP. Echocardiographic assessment revealed diastolic dysfunction in this mouse model, confirming that a single-point mutation in titin can have a measurable effect in whole-heart mechanics in the absence of any other genomic differences. However, no degenerative alterations in the left or right ventricles were observed—thereby not reproducing the human disease phenotype overall. Yet, it is possible that the fibrofatty formations typically manifested at a clinical level in the second or the third decade of life in ARVC patients are not appropriately mimicked in mice. In fact, this appears to be also the case in murine models of other non-titin-based ARVC types (reviewed in [38]). Thus, there is a need for better biological systems to study ARVC. In this respect, new technologies such as iPSC (induced pluripotent stem cell)-derived cardiomyocytes need to be considered. This system has been applied to the study of cardiomyopathies caused by titin truncating mutations [39] and perhaps might be applicable to the study of titin mSNPs. In ARVC, diastolic abnormalities are an early marker of the disease [40], thus the mice model might mimic initial stages of the human pathology. Speculatively, ‘stickiness’ of Ig-tandems in titin's I-band [28,29] (which natively makes little or no connections to the thin filament during the cardiac cycle) would interfere with its compliance, necessary for efficient diastolic filling [41,42]. This could explain the observed phenotype. Finally, there is the possibility that the effect of the T2850I mutation in patients is potentiated by another co-segregating genetic factor, jointly leading to the disease phenotype (e.g. an additive effect of other unknown rare sarcomeric variants). In this regard, more complete genomic analyses will be required in the future.

In conclusion, our results reveal that the contribution of a single mSNP in a generic domain of titin can lead to a measurable alteration of heart function, even though the mSNP might only cause subtle changes at the protein level. Given the staggering number of mSNPs in titin, this finding establishes the high potential of this protein to contribute to disease mechanisms in the heart.

## Supplementary Material

Supplementary Material
