# Supplementary material for: Exploration of pathomechanisms triggered by a single-nucleotide polymorphism in titin's I-band: the cardiomyopathy-linked mutation T2580I
Source: Open Biol. 2016 Sep 28;6(9):160114. doi: 10.1098/rsob.160114 (PMC5043576; doi:10.1098/rsob.160114)
Supplement: Supplementary Material [file rsob160114supp1.pdf]

SUPPLEMENTARY MATERIALS

Exploration of pathomechanisms triggered by a SNP in titin's I-band: the cardiomyopathy-linked mutation T2580I

(Bogomolovas et al., 2016)

Methods

Cloning

A titin gene fragment encoding domains I7-I13 (residues 2560-3189; NCBI protein accession NP_003310) was PCR amplified from a randomly primed cDNA library of human skeletal muscle. The fragment was used as a source of coding sequences for I9-I11 (residues 2749-3009) and I10 (2835-2895). I7-I13, I9-I11 and I10 were inserted into the pETM11 vector using NcoI/Acc65I restriction sites. All constructs carried a His_6-tag and a TEV protease cleavage site N-terminally to the insert. A valine residue was appended to the C-terminus of domain I11 to facilitate crystallization of I9-I11.

Recombinant protein production

Expression used BL21 *E.coli* in TB medium (12 g/L tryptone, 24 g/L yeast extract, 4 mL/L glycerol, 17 mM KH_2PO_4, 72 mM K_2HPO_4). Cells were grown at 37°C to an OD_{600}=1, expression induced with 0.2 mM IPTG, and growth carried out overnight at 18°C. Cells were harvested by centrifugation, washed with Mg^{2+}/Ca^{2+}-free PBS buffer (8g/L NaCl, 0.2g/L KCl, 1.44g/L Na_2HPO_4, 0.24g/L KH_2PO_4), repelleted and stored frozen at -20°C. For protein purification, pellets were resuspended in lysis buffer (25 mM HEPES pH 7.5, 250 mM NaCl, 10 mM Imidazole, 0.2 % NP-40, 2 mM β-mercaptoethanol) supplemented with EDTA-free protease inhibitor cocktail (Roche), 1mg/mL lysozyme, 50 μg/mL DNAse and 5 mM $MgSO_4$. The mixture was then sonicated, centrifuged and the supernatant purified in Ni^{2+}-NTA beads (Qiagen) equilibrated in lysis buffer. Elution used 300 mM Imidazole. Subsequently, samples were incubated with His_6-tagged TEV (Tobacco Etch Virus) protease overnight at room temperature in 25 mM Tris pH 7.5, 50 mM NaCl, 5 mM β-mercaptoethanol. Further purification used subtractive metal affinity in TEV digestion buffer and size exclusion chromatography on a HiLoad 16/60 Superdex 75 PG column in 20 mM HEPES pH 7.3, 50

mM NaCl, 0.5 mM tris(2-carboxyethyl)phosphine (TCEP). The I9-I11 sample was concentrated to 12 mg/mL, I10 to 24 mg/mL and I7-I13 to 3.5 mg/ml and stored at $4^{o}C$ until further use.

For NMR studies, I10 was produced in M9 medium with $^{15}NH_4Cl$ as source of nitrogen and ^{13}C-glucose as carbon source. Protein production was performed as described above, with the exception of gel filtration, which was performed in 20 mM Tris pH 7, 50 mM NaCl, 0.5 mM TCEP, 0.2 % NaN_3. For NMR measurements, the sample was concentrated to 2 mM.

Differential scanning fluorimetry (DSF)

The melting temperature, T_m, of I10 and its variants were determined by DSF. Measurements were performed in triplicate on a Mx3005P Real-Time PCR System (Stratagene). The fluorescence of SYPRO Orange (Invitrogen) was monitored at λ=492nm and λ=610nm for excitation and emission, respectively. Measurements were performed in optical 96-well plates (Applied biosystems). Each well contained 25 µL of sample mixture consisting of 5 µM protein, 15X SYPRO Orange in assay buffer (100 mM HEPES pH 8, 150 mM NaCl). Fluorescence was recorded from 25 to 95 °C with a temperature increase rate of 1 °C/min. The melting profiles recorded were analyzed using MATLAB software as described [1].

Crystal structure determination of I10

I10 crystals were grown in 48-well VDX plates (Hampton Research) at 20 °C in hanging drops consisting of 1 µL protein solution at 24 mg/mL and 1 µL of mother liquor. Crystal grew from two media conditions: (*a*) 0.2 M $CaCl_2$, 30% [w/v] PEG 3350, 0.1 M Tris pH 7.5, 3% [v/v] Isopropanol; (*b*) 0.2 M $CaCl_2$, 25% [w/v] PEG 8000, 0.1 M Tris pH 7.5. Selected crystals from each condition were soaked in mother liquor supplemented with 30% [v/v] glycerol and cryo-cooled in liquid N_2. X-ray diffraction data were processed using XDS/XDSCALE [2] or SCALA [3] for crystal forms *a* and *b*, respectively (**Table S2**).

Phasing was carried out first for crystals from condition *(a)* as they had only 1 molecular copy per asymmetric unit. Phases were calculated with Phaser [4], using as a search model for molecular replacement (MR) a preliminary NMR structure of I10 (calculated within this work). The crystallographic model was manually rebuilt in Coot [5] and refined using

phenix.refine [6]. This structure was then used as search model in MR to phase the data from crystals grown in condition *b*, which contained 16 copies per asymmetric unit. Model refinement was as above, but included automatic model building in ARP/wARP [7].

Crystal structure determination of I19-I11

Crystals grew in sitting drops consisting of 200 nL of protein solution (12-16 mg/mL) and 200 nL of mother liquor in 96-well Intelliplates (Art Robbins Instruments) at 19 °C. Crystals were obtained from two media conditions: (*c*) 0.1 M Tris HCl pH 8.5, 30% [w/v] PEG 4000, 0.2 M $MgCl_2$; (*d*) 0.1 M Bis-Tris Propane pH 8.5, 20% [w/v] PEG 3350, 0.2 M sodium acetate. For data collection, crystals were soaked in mother liquor supplemented with 20% [v/v] glycerol and cryo-cooled in liquid N_2. Data processing used iMosflm [8] and SCALA [3] (**Table S2)**. Phasing of data from condition *c* was by MR in Phaser [4] using one other Ig domain of titin as search model (PDB ID 1WAA). Automatic model building used ARP/wARP [7]. Manual building was in Coot [5] and refinement in PHENIX [6] with each Ig domain treated as a TLS group.

The crystal structure of I9-I11 in condition *d* was solved by MR using the crystal structure of I9-I11 from *c* as search model. The position of individual Ig was adjusted by rigid body refinement in PHENIX [6] and the model refined as before.

NMR studies of I10 and I10^{T2850I}

NMR spectra were acquired at 22 °C on Bruker DRX500, Avance III 600 and Avance III 700 spectrometers. All spectra were processed with nmrpipe and analyzed with Nmrview [9]. Sequential backbone resonances (NH, N, CO, Cα, Cβ) were assigned from the following 3D experiments: HNCO, HNCA, HNCACB, CACB(CO)NH. ^{15}N T1, T2 and heteronuclear NOE data were acquired with an interleaved data acquisition scheme at 500 MHz.

The $^3J\ C'_{k-1}H\alpha_k$ values were determined from a CO-coupled 3D(H)NCαHα experiment [10]. J-coupling values were extracted from processed spectra using Nmrview [9] and related to the main chain ϕ angles using the Karplus equation [11]:

$$(3.62 * \cos((x - 60) * \frac{\pi}{180})^2 + 2.11 * \cos((x - 60) * \frac{\pi}{180}) + 1.29).$$

Molecular dynamics simulations

The crystal structure of I10-I11 was used as wild-type in MDS. Two versions of I10-I11^{T2850I} were modelled by replacement of T2850 with isoleucine, using a different rotamer in each case. The models were placed in the center of a rhombic dodecahedran water box (at 1.0 nm from the box edge) and counterions added to neutralize the system. To assess whether MDS parameters were appropriate to sample the amplitude of the interdomain motion in I10-I11, I65-I66 from titin (PDB ID 3B4B) was used as control. Simulations were performed using GROMACS package 5.0 [12] with the AMBER99SB-ILDN forcefield [13]. The TIP3P and SPC/E water models were used comparatively and showed not to influence the conclusion drawn. An initial energy minimization of all systems was performed using steepest descents algorithm. The Particle Mesh Ewald (PME) algorithm [14] was used for long-range electrostatics with non-bonded and van der Waals cut-offs of 1nm. All bond lengths were constrained using the LINCS algorithm [15]. Calculations used a constant temperature of 300K, a pressure of 1 bar, an integration time step of 2 fs and periodic boundary conditions. A final MD run of 50 ns was performed for all systems. Principal component analysis was performed on the concatenated backbone trajectories using the GROMACS modules g_convar and g_anaieg. PDB2PQR [16] and APBS [17] were used to calculate Poisson–Boltzmann electrostatic surfaces. Results were visualized in PyMol v.1.5.0.4 (Schrödinger LLC) using an APBS scale of -5 kT/e to +5 kT/e.

Transgenic muscle

For *in vivo* visualisation, wild-type and mutant I7-I13 fragments were cloned into the pEGFP-C1 vector, resulting in N-terminal fusion with eGFP. Transfection of GFP tagged I-band titin fragments into *tibialis anterior* muscles was done as previously described [18]. Mice were anesthetized with an intraperitoneal injection of Rompun (Bayer Pharma; active substance ketamine; 5mg/kg) plus Zoletil 100 (Vibrac Laboratories, active substances tiletamine and zolazepam; 30mg/kg). After hair removal, the skin and fascia overlying the *tibialis anterior* were incised. One electrode plate was inserted underneath the muscle, 10 µg of expression plasmid were injected into the *tibialis anterior* muscle and a second electrode plate was placed above the muscle. Five 25 V and 20 ms long electric pulses were applied (ECM 830; BTX Genetronics). The electrodes were removed, and the wound was closed. Ten days later, mice were euthanized by cervical dislocation and transfected muscle sections were

counterstained with Texas Red®-X Phalloidin and visualized under a laser scanning confocal microscope.

Neonatal mouse cardiomyocytes

Isolation and transfection was performed as previously described [19]. Briefly, 1-3 days old neonatal mice were used for cardiomyocyte isolation. Excised hearts were washed with calcium and magnesium free PBS supplemented with 20 mM 2,3-butanedione monoxime (BDM), and pre-digested overnight with 0.0125% trypsin in calcium and magnesium free HBSS supplemented with 20mM BDM at 4 °C. The next day, the trypsin solution was removed, and the cells were isolated from the tissue by collagenase/dispase (Roche) treatment at 37 °C, twice for 20 min. Isolated cells were collected through a cell strainer, centrifuged, re-suspended in plating medium (65% DMEM high glucose, 19% M-199, 10% horse serum, 5% fetal calf serum, 1% penicillin/streptomycin) and pre-plated into an uncoated cell-culture dish. After 2 hours incubation in a cell-incubator, non-adherent cardiomyocytes were collected and finally plated onto collagen-coated cell-culture dishes at a density of approximately 1.5 x 10^5 cells per cm^2. After overnight incubation, cells were changed into Opti-Mem (Thermo Fisher Scientific), and transfected with either GFP tagged wildtype or mutant I7-I13 fragments using Escort III (Sigma-Aldrich) according manufacturer recommendations. 24-hours post transfection, cells were hung in maintenance medium (78% DMEM high glucose, 17% M-199, 4% horse serum, 1% penicillin/streptomycin) and cultured for another 24 hours before fixation or isoproterenol treatment. Cells undergoing isoproterenol stimulation were changed into maintenance medium supplemented with isoproterenol (1µM) or vehicle (water), and cultured for additional 6 hours before fixation. After culturing, cells were fixed with 4% paraformaldehyde in PBS, and subsequently processed for immunofluorescence as described [20]. Cells were counterstained with an antibody against α-actinin (clone EA53, A7811, Sigma-Aldrich), filamentous actin (fluorescent phalloidin, Molecular Probes) and DNA (DAPI, Sigma-Aldrich) following standard protocols and imaged using a confocal microscope (Olympus). Images were processed using ImageJ with the Bio-Formats plugin, and Photoshop (Adobe).

Mouse model generation and phenotyping

A gene targeting strategy was employed to introduce an ACC to ATC point mutation in exon 37 of the mouse titin gene in order to mimic the T2850I human mutation. The targeting vector was built using a vector backbone carrying neomycin resistance for a positive

selection. The targeting vector consists of two arms of homology. The short arm of homology was 3.3 kb in size and carries the point mutation, and the long arm of homology was 4.1 kb. The point mutation was introduced using a site-directed mutagenesis kit (Agilent, Santa Clara, CA) and its presence was confirmed by sequencing. The targeting vector DNA was electroporated into 129S6 embryonic stem cells. The neomycin resistant clones were screened by PCR using primers outside the homologous arms and neomycin primers in order to select the clones that have taken the targeting DNA by homologous recombination. Mice carrying the mutation were genotyped by PCR across the mutation, using a forward primer on the 5' end of the mutation and a neo reverse primer. The PCR amplified bands were gel purified and sequenced to confirm the presence of the mutation. The mice used in this study were on a mixed background, 50% 129S6 and 50% C57BL/6.

For genotyping, mouse tails were digested in tail lysis buffer and incubated at 55° C overnight. Primers were added and a standard PCR cycling protocol performed. PCR products were determined by capillary electrophoresis using an Applied Biosystems 3730 DNA Analyzer, with the wildtype amplicon at 253 bp and the T2850I knock-in (KI) amplicon at 330 bp.

Next, mice were echoed under 1.0% isoflurane anesthesia (USP, Phoenix) in 100% oxygen. Transthoracic echo images were obtained with a Vevo 2100 High Resolution Imaging System (Visual-Sonics, Toronto) using the model MS250 scan head designed for murine cardiac imaging. Care was taken to avoid animal contact and excessive pressure. Images were collected and stored as digital cine loops for offline analysis (Vevo 2100 software suite). Standard imaging planes, m-mode, Doppler, and functional calculations were obtained according to American Society of Echocardiography guidelines [21]. The short-axis view was used for m-mode acquisition of percent fractional shortening and ventricular geometry. Left atrium dimensions were measured in the parasternal long-axis view directly below the aortic valve leaflets. Passive left ventricle filling peak velocity, atrial contraction flow peak velocity, and E and A wave velocities were determined from the images of mitral valve Doppler flow from the apical four-chamber view. Mouse heart rates were maintained between 500-550 bpm for M-mode and 350-450 bpm for Doppler studies.

Statistical analysis used two-way ANOVAs with repeated measures or Tukey's multiple comparisons post-hoc test, paired t-tests, and unpaired t-tests were performed where noted. $P < 0.05$ is considered significant.

Section S1: Thermostability of the I10 domain and its variants

To estimate the effect of the T2850I exchange on the fold stability of I10, the thermal denaturation of wild-type I10^{wt} and I10^{T2850I} was measured by DSF. A truncated I10 missing the N-terminal β-strand A is included here. The findings reveal that the T2850I exchange is as destabilizing as the removal of a small secondary structure element.

Figure S1: DSF analysis of I10.

a. Melting curves of wild-type and mutant I10 proteins; **b**. Crystal structure of I10 and N-terminal sequences of the domain highlighting the differences between the full-length domain and the N-terminally truncated version; **c.** Summary Table of Tm values measured by DSF.

a.

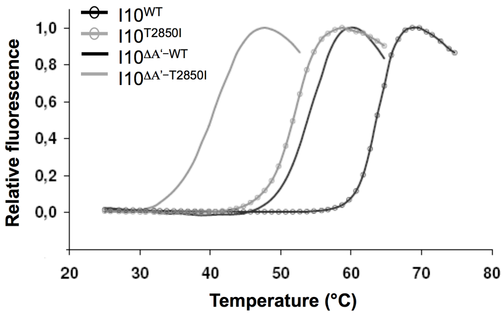

b.

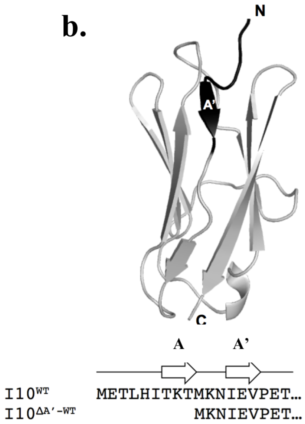

c.

Tm values

I10^{wt} → 62.3 ± 1.3 ^{o}C
I10$^{\Delta A\text{-}wt}$ → 52.3 ± 1.3 ^{o}C
I10^{T2850I} → 51.4 ± 1.8 ^{o}C
I10$^{\Delta A\text{-}T2850I}$ → 38.4 ± 1.8 ^{o}C

Section S2: X-ray Crystallography

Table S2a: Data statistics and model refinement parameters of the crystal structures of I10 and I9-I11

	**I10** (*a*)	**I10** (*b*)	**I9-I11** (*c*)	**I9-I11** (*d*)
PDB accession code	4QEG	5JDJ	5JDE	5JDD
Space group	$P4_1$	$P2_12_12_1$	P1	$P2_12_12_1$
Cell dimensions:<br>*a*, *b*, *c* (Å)<br>α, β, γ (°)	31.2, 31.2, 93.9<br>90, 90, 90	74.6, 136.2, 140.1<br>90, 90, 90	37.7, 42.4, 83.5<br>85.2, 79.8, 87.0	42.3, 65.8, 108.4<br>90, 90, 90
Solvent content (%)	51	46	44	52
Matthews coefficient ($Å^3$/Da)	2.49	2.27	2.17	2.52
Copies in asymmetric unit	1	16	2	1
**X-ray data**				
X-ray source	DIAMOND I24	DIAMOND I04-1	DIAMOND I02	DIAMOND I04
Detector	PILATUS 6M	PILATUS 2M	PILATUS 6M	PILATUS 6M-F
Wavelength (Å)	0.9464	0.9173	0.9795	0.9795
Detector-crystal distance (mm)	502	222	347	285
Resolution (Å)	26.00-2.00 (2.071-2.00)	29.65-1.738 (1.80-1.738)	19.427-1.906 (1950-1.906)	41.83-1.53 (1.610-1.530)
Unique reflections	5665 (560)	145378 (13857)	38254 (6392)	45488 (6392)
R_{sym} (I) (%)	13.9 (34.13)	9.6 (71.4)	7.6 (64.5)	5.8 (41.4)
Multiplicity	2.2 (1.9)	7.5 (7.1)	3.5 (3.4)	5.5 (5.1)
Completeness, %	93.2 (91.95)	98.77 (95.19)	96.61 (86.2)	97.5 (95.9)
I/σ (I)	4.96 (2.38)	15.38 (2.77)	11.3 (1.8)	12.6 (3.8)
**Refinement**				
No. of reflections Working/test set	5215/449	143915/1455	38230/1151	45171/2687
Protein atoms/solvent molecules	696/123	11199/1625	4089/264	2131/543
Ligands	3 x Ca^{2+}	-	-	-
Rfactor/Rfree, %	17.53/22.99	17.45/20.49	19.28/22.83	16.15/19.65
RMSD bond length, Å	0.007	0.007	0.08	0.006
RMSD bond angle, °	1.092	1.16	1.119	1.094

Figure S2: Electron density map of the β-turn A'B from I10

(2mFo-DFc)αc electron density map contoured at 1.5σ

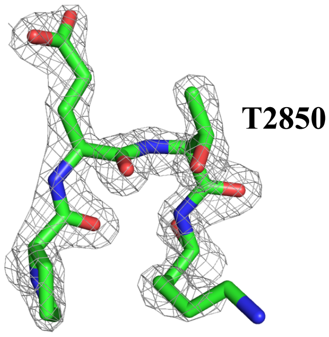

Table S2b: Directed Ig-Ig interactions in the crystal structures of I9-I11

The contacts are detailed for each of the molecular copies calculated in this study. Namely, one copy in space group $P2_12_12_1$ and two non-crystallographic copies (A and B) in space group P1.

I9			I10			Distance		
Nr	residue	atom	Nr	residue	atom	$P2_12_12_1$	*P*1 A	*P*1 B
166	ASN	ND2	17	GLU	OE2	3.82	4.86	4.68
89	GLU	OE1	116	HIS	NE2	2.59	2.46	2.65
90	THR	O	92	HIS	ND1	2.87	3.41	2.85
90	THR	O	92	HIS	N	3.44	4.56	3.34
90	THR	O	115	SER	OG	3.55	3.69	3.47

I10			I11			Distance		
Nr	residue	atom	Nr	residue	atom	$P2_12_12_1$	*P*1 A	*P*1 B
104	GLU	OE1	178	ILE	N	2.85	2.91	3.64
104	GLU	OE1	253	LYS	N	3.24	4.03	3.25
104	GLU	OE2	253	LYS	N	3.24	3.28	3.52
105	THR	OG1	207	SER	N	2.99	3.11	3.75
176	THR	O	178	ILE	N	3.65	4.63	3.54
176	THR	OG1	203	TYR	OH	3.49	2.37	2.67
177	PRO	O	179	MET	N	3.5	3.74	N/A
177	PRO	O	202	ASN	ND2	3.87	2.99	N/A
106	LYS	NZ	204	GLU	O	2.94	3.26	3.45

Section S3: Analysis of type II β-turns composition in proteins

Using PDBeMotif (http://www.ebi.ac.uk/pdbe-site/pdbemotif/; [22]) and upon removal of redundant protein entries, we identified 62,641 β-turns of type II in protein structures deposited with the Protein Data Bank repository. The analysis of turn composition is shown below. Those exceptional cases where proline or isoleucine residues were found in position *i+2* were examined manually and found to correspond to highly unusual turns inserted into hydrophobic pockets.

Table S3: Residue composition in a total of 62,641 β-turns of type II in proteins

Residue	*i+1*	*i+2*
A	6428	639
C	411	315
D	2911	2348
E	5851	602
F	1455	564
G	948	48760
H	996	765
I	1468	**2**<br>(PDB 3ZPX:A148-151)<br>(PDB 4JOS:A112-115)
K	6227	594
L	2207	373
M	682	280
N	1679	4176
P	15199	**1**<br>(PDB 1RLM:C266-269)
Q	2681	543
R	2669	807
S	4271	741
T	2028	272
V	2750	22
W	459	147
Y	1321	690

Figure S3: Stability of all possible T-to-aa replacements

Energy changes (ΔΔG) for the replacement of T2850 by every other amino acid calculated using FoldX [23]. The values confirm other observations in this work that Pro, Ile and Val residues are particularly poorly tolerated at this position leading to the destabilization of the I10 domain.

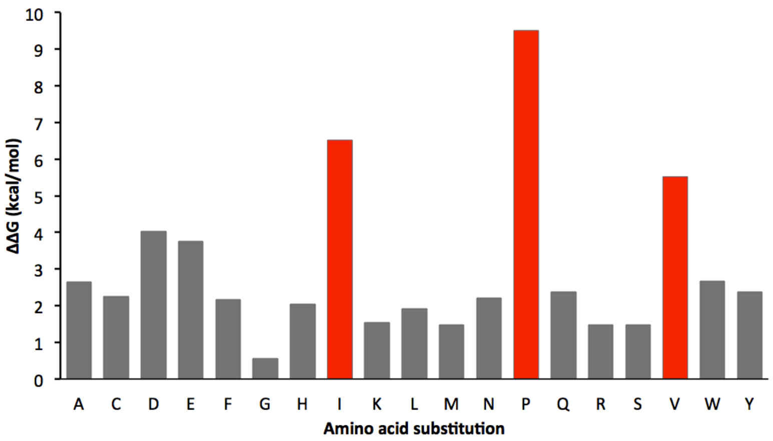

Section S4: Molecular Dynamics Simulations on I10-I11

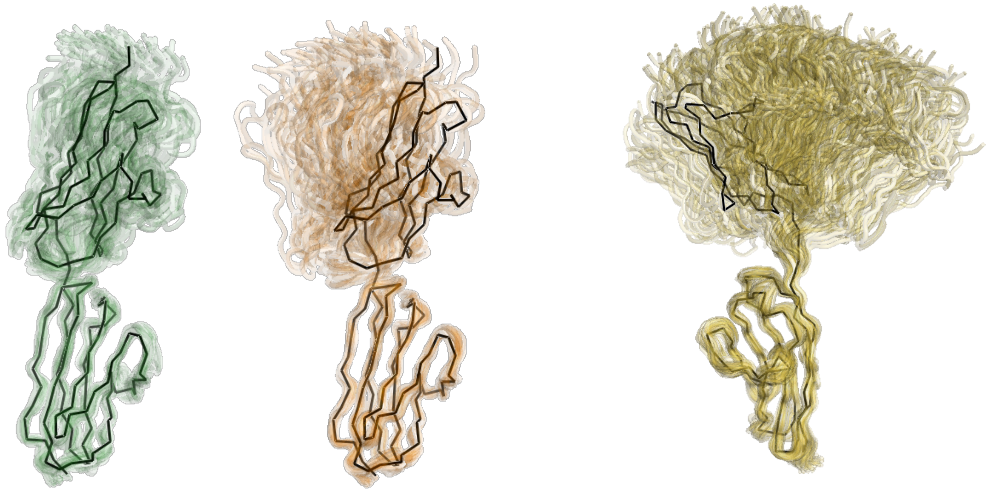

wt T2850I I64-I65

Figure S4a: Spread of the motion amplitude for test and control structures.
Cartoon representation of conformers sampled every 0.5 ns. Original crystal structure shown as black ribbon.

Figure S4b: Principal component (PC) analysis of domain movements

The motion is decomposed and described as movement along a series of vectors. The PC values describe the linear position along each vector and, therefore, the position between two extremes conformations. In this study of I10-I11, the first 10 eigenvalues were analyzed and found that the first 3 describe most movements. The other vectors describe smaller movements such as loop movements

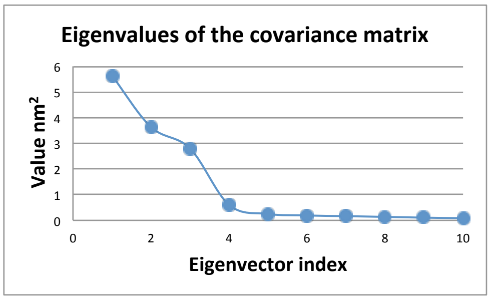

Figure S4c: Progression of Principal component values throughout the simulation

Eigenvalues plotted against frame number (1 frame = 2 fs) with LOESS (locally weighted scatterplot smoothed) lines; wildtype, blue; T2850I mutant, red. The plots show that the domain movement converges during the simulation and, therefore, that the duration of the simulations was appropriate. It can also be seen that only PC1 shows a noticeable difference between the wild-type I10-I11 and the T2850I variant.

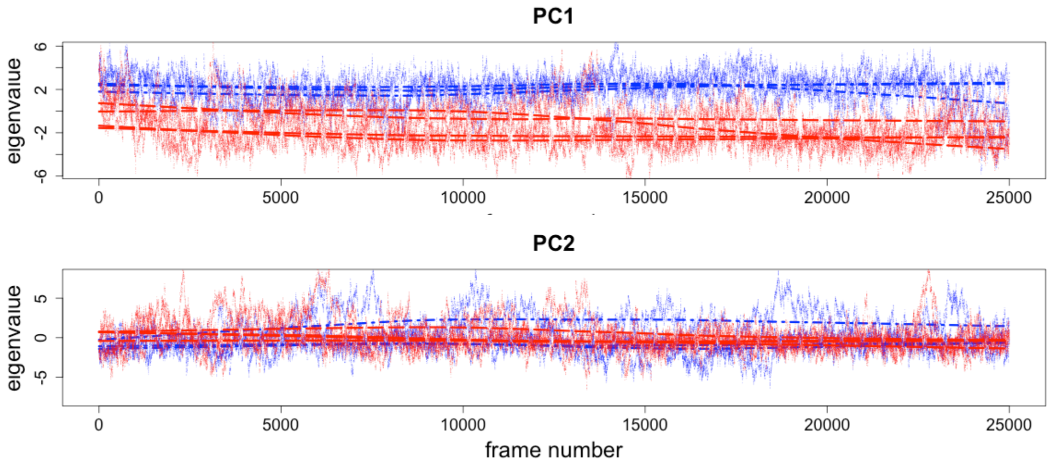

Figure S4d: Illustration of principal components

Two conformations of I10-I11 resulting from MDS, corresponding to the smallest (orange) and largest (cyan) eigenvalue. Conformers are superimposed on the I10 domain so that the variation in I11 can be appreciated. For clarity, a plane is shown through I11 in each conformer. **a.** Top view along the extreme eigenprojections of PC1. **b.** Side view showing the extreme eigenprojections of PC2. Eigenprojections do not necessarily represent actual protein conformations occurring in the trajectories as conformations are a combination of all components of movement.

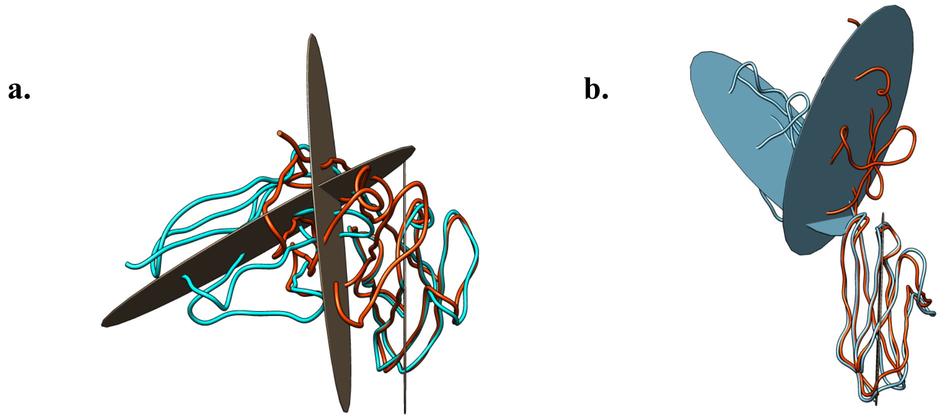

Section S5: Evaluation of the association of the I7-I13 titin fragment with myocellular structures

S5a: Subcellular fractionation of transgenic muscle

For western blot analysis transgenic muscle extracts were fractionated as described earlier [24] with minor modifications and probed for GFP using anti-GFP (abcam ab290) antibody as described [25]. Briefly, tissue was finely minced using scissors and washed in cold PBS. Tissue was homogenized using glass-teflon homogenizer in homogenization buffer (250 mM sucrose, 50 mM Tris-HCl (pH 7.4), 5 mM $MgCl_2$) supplemented with 1 mM DTT 1 mM, 25 μg/ml spermine, 25 μg/ml spermidine and EDTA-free protease inhibitor cocktail (Roche). Particulate material consisting mainly of nuclei, mitochondria and myofibrils was pelleted by spinning at 6000 *g* for 15 min in cooled centrifuge. To pellet microsomal fraction

supernatant was supplemented with 8 mM $CaCl_2$, incubated on ice for 10 minutes and spun down for 15 minutes at 9000 *g*. The remaining supernatant contained cytosol components.

Figure S5a: Subcellular fractionation of transgenic muscle

Wester blot (left) and Coomasie SDS-PAGE (right) are shown.

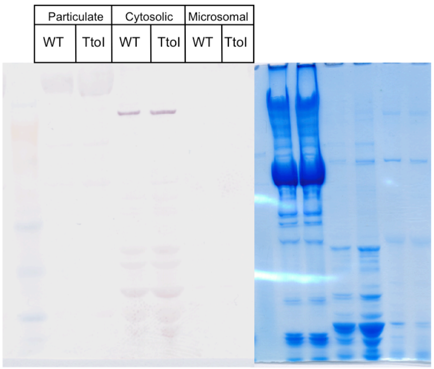

S5b: Actin co-sedimentation experiments

An actin co-sedimentation assay was performed as described previously [26, 27]. G-actin prepared from rabbit back muscle was centrifuged at 100,000 *g* for 30 min and the supernatant polymerised in 50 mM NaCl, 2 mM Tris-HCl, pH 7.5, 1 mM $MgCl_2$, 1 mM DTT. Purified titin fragments were centrifuged at 100,000 *g* for 30 min immediately before the assay to sediment any insoluble protein. Titin fragments (20 μM) were added to polymerized F-actin (10 μM) in a total volume of 100 μl. Samples were incubated for 1 h at room temperature and centrifuged at 100,000 *g* for 30 min. Supernatant and pellet fractions were separated by SDS-PAGE.

Figure S5b: Actin co-sedimentation. No detectable interaction between actin and wild-type or mutated I7-I13 titin fragment was seen in Coomassie stained SDS PAGE. Titin fragments remained in the supernatant fraction, whereas actin was found in the pellet.

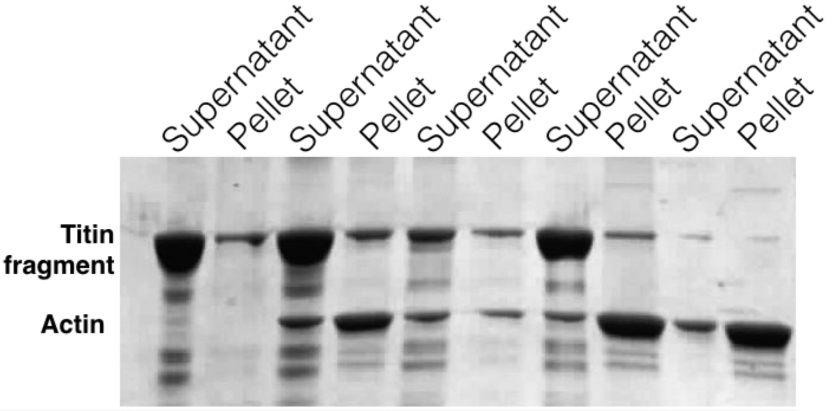

Actin	-	+	-	+	+
I7-I13^{WT}	+	+			
I7-I13^{T2850I}			+	+	

Figure S5c: Low resolution images of transgenic muscle

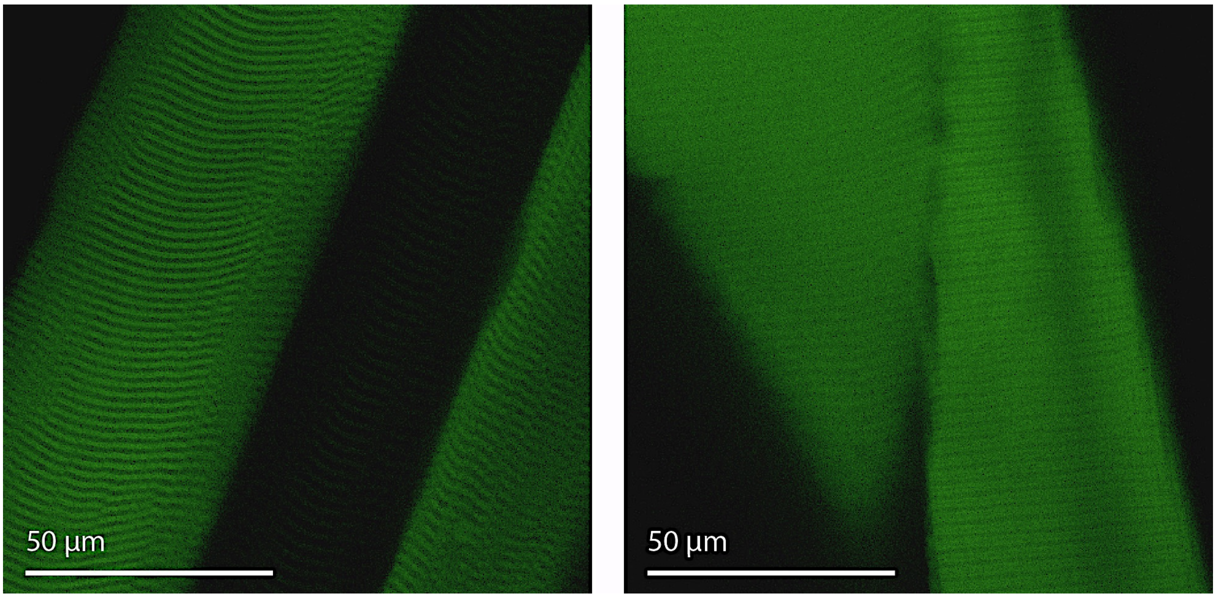

I10-T2850I **wild-type I10**

References

1 Matulis D, Kranz JK, Salemme FR, Todd MJ. 2005 Thermodynamic stability of carbonic anhydrase: measurements of binding affinity and stoichiometry using ThermoFluor. *Biochemistry.* **44**, 5258-5266.

2 Kabsch W. 2010 XDS. *Acta Crystallogr. D Biol. Crystallogr.* **66**, 125-132.

3 Evans P. 2006 Scaling and assessment of data quality. *Acta Crystallogr. D Biol. Crystallogr.* **62**, 72-82.

4 McCoy AJ, Grosse-Kunstleve RW, Adams PD, Winn MD, Storoni LC, Read RJ. 2007 Phaser crystallographic software. *J. Appl. Crystallogr.* **40**, 658-674.

5 Emsley P, Lohkamp B, Scott WG, Cowtan K. 2010 Features and development of Coot. *Acta Crystallogr. D Biol. Crystallogr.* **66**, 486-501.

6 Adams PD, Afonine PV, Bunkóczi G, Chen VB, Echols N, Headd JJ, Hung LW, Jain S, Kapral GJ, Grosse Kunstleve RW, McCoy AJ, Moriarty NW, Oeffner RD, Read RJ, Richardson DC, Richardson JS, Terwilliger TC, Zwart PH. 2011 The Phenix software for automated determination of macromolecular structures. *Methods.* **55**, 94-106.

7 Langer G, Cohen SX, Lamzin VS, Perrakis A. 2008 Automated macromolecular model building for X-ray crystallography using ARP/wARP version 7. *Nat. Protoc.* **3**, 1171-1179.

8 Battye TG, Kontogiannis L, Johnson O, Powell HR, Leslie AG. 2011 iMOSFLM: a new graphical interface for diffraction-image processing with MOSFLM. *Acta Crystallogr. D Biol. Crystallogr.* **67**, 271-281.

9 Johnson BA. 2004 Using NMRView to visualize and analyze the NMR spectra of macromolecules. *Methods Mol. Biol.* **278**, 313-352.

10 Löhr F, Rüterjans H. 1995 (H)NCAHA and (H)CANNH experiments for the determination of the vicinal coupling constants related to the phi-torsion angle. *J. Biomol. NMR.* **5**, 25-36.

11 Wang AC, Bax, A. 1996 Determination of the Backbone Dihedral Angles φ in Human Ubiquitin from Reparametrized Empirical Karplus Equations. *J. Am. Chem. Soc.* **118**, 2483-2494.

12 Van Der Spoel D, Lindahl E, Hess B, Groenhof G, Mark AE, Berendsen HJ. 2005 GROMACS: fast, flexible, and free. *J. Comput. Chem.* **26**, 1701-1718.

13 Lindorff-Larsen K, Piana S, Palmo K, Maragakis P, Klepeis JL, Dror RO, Shaw DE. 2010 Improved side-chain torsion potentials for the Amber ff99SB protein force field. *Proteins.* **78**, 1950-1958.

14 Darden T, Perera L, Li L, Pedersen L. 1999 New tricks for modelers from the crystallography toolkit: the particle mesh Ewald algorithm and its use in nucleic acid simulations. *Structure.* **7**, R55-60.

15 Berk H, Bekker H, Berendsen HJC, Fraaije JGEM. 1997 LINCS: a linear constraint

solver for molecular simulations *J. Comp. Chem.* **18**, 1463-1472.

16 Dolinsky TJ, Czodrowski P, Li H, Nielsen JE, Jensen JH, Klebe G, Baker NA. 2007 PDB2PQR: expanding and upgrading automated preparation of biomolecular structures for molecular simulations. *Nucleic Acids Res.* **35**, W522-525.

17 Baker NA, Sept D, Joseph S, Holst MJ, McCammon JA. 2001 Electrostatics of nanosystems: application to microtubules and the ribosome. *Proc. Natl. Acad. Sci. USA.* **98**, 10037-10041.

18 Donà M, Sandri M, Rossini K, Dell'Aica I, Podhorska-Okolow M, Carraro U. 2003 Functional in vivo gene transfer into the myofibers of adult skeletal muscle. *Biochem. Biophys. Res. Commun.* **312**, 1132-1138.

19 Ehler E, Moore-Morris T, Lange S. 2013 Isolation and culture of neonatal mouse cardiomyocytes. *J. Vis. Exp.* **79**, 50154.

20 Lange S, Perera S, Teh P, Chen J. 2012 Obscurin and KCTD6 regulate cullin-dependent small ankyrin-1 (sAnk1.5) protein turnover. *Mol. Biol. Cell.* **23**, 2490-504.

21 Gardin JM, Adams DB, Douglas PS, Feigenbaum H, Forst DH, Fraser AG, Grayburn PA, Katz AS, Keller AM, Kerber RE, Khandheria BK, Klein AL, Lang RM, Pierard LA, Quinones MA, Schnittger I. 2002 Recommendations for a standardized report for adult transthoracic echocardiography: A report from the American society of Echocardiography's nomenclature and standards committee and task force for a standardized Echocardiography report. *J.Am. Soc. Echocard.* **15**, 275-290.

22 Golovin A, Henrick K. 2008 MSDmotif: exploring protein sites and motifs. BMC *Bioinformatics.* **9**, 312.

23 Van Durme J, Delgado J, Stricher F, Serrano L, Schymkowitz J, Rousseau F. 2011 A graphical interface for the FoldX forcefield. *Bioinformatics*. **27**, 1711-1712.

24 Cox B, Emili A. 2006 Tissue subcellular fractionation and protein extraction for use in mass-spectrometry-based proteomics. *Nat. Protoc.* **1**, 1872-1878.

25 Moriscot AS, Baptista IL, Bogomolovas J, Witt C, Hirner S, Granzier H, Labeit S. 2010 MuRF1 is a muscle fiber-type II associated factor and together with MuRF2 regulates type-II fiber trophicity and maintenance. *J. Struct. Biol.* **170**, 344-353.

26 Srivastava J, Barber D. 2008 Actin co-sedimentation assay; for the analysis of protein binding to F-actin. *J. Vis. Exp.* **13**, 690.

27 Spudich JA, Watt S. 1971 The regulation of rabbit skeletal muscle contraction. I. Biochemical studies of the interaction of the tropomyosin-troponin complex with actin and the proteolytic fragments of myosin. *J. Biol. Chem.* **246**, 4866–4871.
